# Lightweight helmet target detection algorithm combined with Effici-Bi-Level Routing Attention

**DOI:** 10.1371/journal.pone.0303866

**Published:** 2024-05-29

**Authors:** Yanguo Huang, Minjie Fang, Jian Peng

**Affiliations:** College of Electrical and Automation, Jiangxi University of Science and Technology, GanZhou, China; University of North Florida, UNITED STATES

## Abstract

Wearing helmets is essential in two-wheeler traffic to reduce the incidence of injuries caused by accidents. We present FB-YOLOv7, an improved detection network based on the YOLOv7-tiny model. The objective of this network is to tackle the problems of both missed detection and false detection that result from the difficulties in identifying small targets and the constraints in equipment performance during helmet detection. By applying an enhanced Bi-Level Routing Attention, the network can improve its capacity to extract global characteristics and reduce information distortion. Furthermore, we deploy the AFPN framework and effectively resolve information conflict using asymptotic adaptive feature fusion technology. Incorporating the EfficiCIoU loss significantly improves the prediction box’s accuracy. Experimental trials done on specific datasets reveal that FB-YOLOv7 attains an accuracy of 87.2% and 94.6% on the mean average precision (mAP_@.5_). Additionally, it maintains a high level of efficiency with frame rates of 129 and 126 frames per second (FPS). FB-YOLOv7 surpasses the other six widely-used detection networks in terms of detection accuracy, network implementation requirements, sensitivity in detecting small targets, and potential for practical applications.

## 1 Introduction

Recently, due to the swift advancement of new energy technology, electric bicycles and other two-wheeled vehicles have gained popularity among individuals. This is mostly due to their energy efficiency, environmental friendliness, and ease, making them the preferred mode of transportation for both personal travel and distribution services. Nevertheless, as the frequency of use increases, traffic congestion and accidents are also increasing. In China, the absence of organised driving instruction and evaluation has led to a lack of knowledge among cyclists regarding traffic safety. Consequently, the mortality rate for bicycle and electric bicycle accidents has risen from 4.86% in 2000 to 6.97% in 2019 [1]. Meanwhile, in the United States, the fatality rate for motorcyclists in traffic accidents is 28 times higher than that of passengers in cars, a statistic that will reach concerning levels in 2020 [2]. Brain injury is the primary cause of the majority of these accidents are caused by brain injuries. Research has demonstrated that wearing a helmet properly can decrease the likelihood of a head injury by 60% and the death rate by 71% [3]. Hence, the surveillance of helmet usage plays a crucial role in mitigating fatalities from road accidents and enhancing drivers’ consciousness of safety, making it an imperative issue that cannot be disregarded.

Computer vision and machine learning have made substantial advancements in road traffic analysis in recent years. Algorithms based on traditional methods and deep learning primarily categorize the approaches for target detection. People commonly employ the deep learning technique because of its effective feature extraction and high detection rate. The target detection algorithm has evolved into two categories: One-Stage and Two-Stage. The former, such as the YOLO [4] series and SSD [5], have a fast detection rate and are suited for meeting the demands of road traffic. The latter, such as R-CNN [6] and Fast R-CNN [7], have accurate recognition but slower speed and a more complex network. Consequently, the One-Stage approach is often preferred for road traffic detection.

Currently, the majority of scholars researching helmet detection employ the One-Stage method and seek to optimize it. Wu et al. [8] improved detection performance by replacing the YOLO v3 [9] backbone with Densenet [10]. Jin et al. [11] modified the output of the YOLOv4 [12] feature map to 4, added a 128 × 128 feature map output, and improved the feature fusion module to achieve feature reuse, thus obtaining better classification results. Xue et al. [13] enhanced the quality of retrieved features by integrating channel and spatial attention-weighted features with dense connection networks. On the other hand, Jia et al. [14] substantially increased the accuracy of the model by including the attention mechanism in the YOLOv5 method and utilizing the Soft-NMS [15] algorithm. In 2021, Lv [16] used the CenterNet [17] algorithm with the HOI (Human Object Interaction) to achieve real-time and precise detection of motorcyclists’ safety helmet usage through comprehensive labeling.

Despite the present optimisation strategy enhancing the accuracy of helmet detection, there remain unresolved issues. Due to its small size, the helmet will cause accidents of missed detection and false detection due to the influence of many targets, occlusion, illumination, angle and other factors in the monitoring screen. Furthermore, given the constrained capabilities of edge terminal devices, it is crucial to strike a balance between the precision of detection and the processing resources required for helmet detection tasks.

To address the aforementioned issues, we suggest the implementation of the FB-YOLOv7 network, which builds upon the enhancements made to YOLOv7-tiny. This paper’s main contributions are as follows:

1. A lightweight FB-YOLOv7 network is proposed, which combines the One-Stage algorithm with self-attention mechanism. It also adds the AFPN structure and optimises the loss function. This network’s purpose is to identify helmet use and vehicles in difficult road environments.
2. In order to save computing resources, E-BRA is proposed to improve global search efficiency by filtering out regions with low correlation.

The subsequent sections of this article are organised in the following manner: “Section 2: Method”provides a comprehensive explanation of the network presented in this paper. The empirical findings are presented in “Section 3: Experiment Results”. And draw a conclusion in “Section 4: Conclusion”.

## 2 Method

### 2.1 YOLOv7-tiny

**Fig 1:**
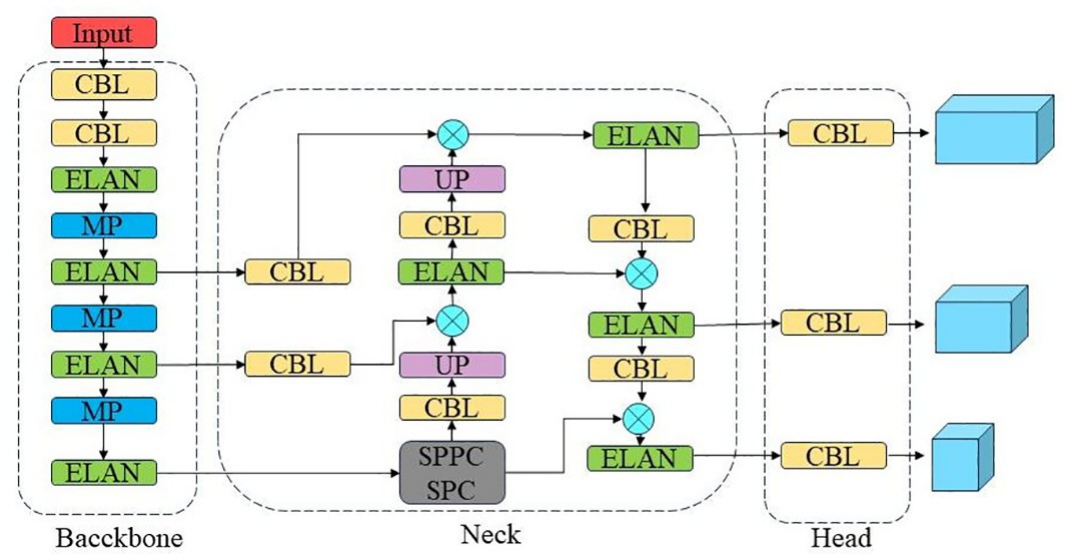
YOLOv7-tiny network structure.

YOLOv7 is a target detection network model introduced by Wang et al. in 2022 [18]. Within the FPS range of 5 to 160, the YOLOv7 network demonstrates significant superiority in terms of speed and accuracy compared to existing One-Stage algorithms. Specifically designed for edge terminal devices, YOLOv7-tiny is a network model. It is based on YOLOv7 and consists of three primary components: Backbone, Neck, and Head. Fig 1 displays the structure.

In the Backbone section, a more compact ELAN is utilised instead of an E-ELAN for feature extraction, while MPConv is maintained for downsampling. In the Neck section, the features processed by SPPCSPC are combined using the PANet [19] structure. Lastly, the Head section employs the RepConv [20] module to enhance inference speed and generate prediction results of three distinct sizes.

### 2.2 FB-YOLOv7

This paper introduces the FB-YOLOv7 network, which aims to enhance the detection accuracy of helmet-wearing states and vehicles while minimising the chances of missing small targets. The network is an optimised version of YOLOv7-tiny, focusing on improving feature extraction, spatial feature fusion, and loss function.

**Fig 2:**
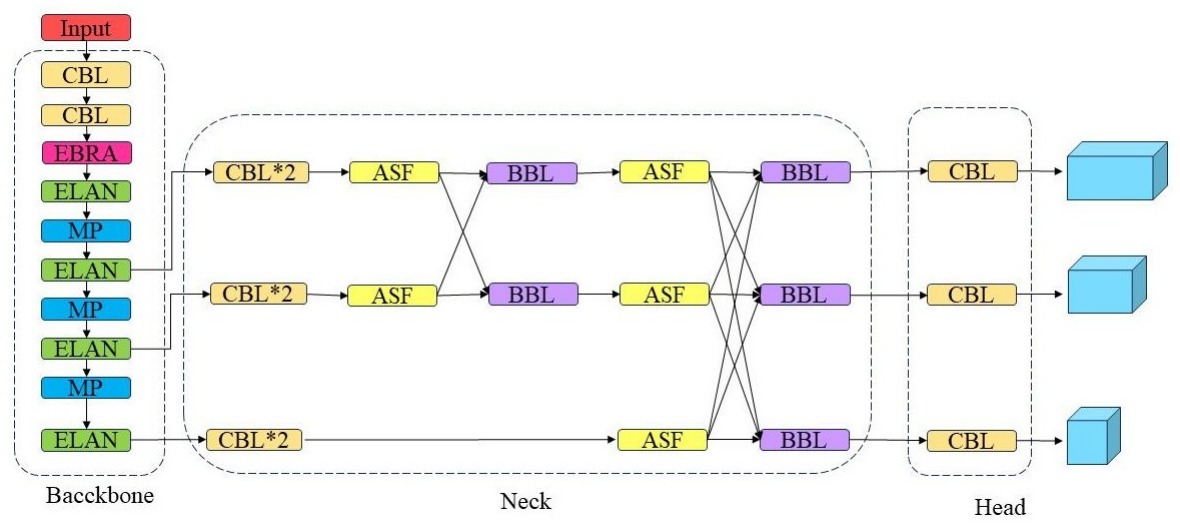
FB-YOLOv7 network structure.

The main improvement method is to add the E-BRA module to the process of extracting features. By excluding the low correlation zone, attention is focused on the high correlation region, allowing for accurate feature extraction while minimising computational resources. The feature pyramid has been improved to AFPN, and asymptotic adaptive spatial feature fusion is employed to preserve the information of low-level features and minimise the potential for information conflict. We have modified the loss function to ECIoU to enhance the network’s resilience and responsiveness towards small targets. In the subsequent sections, we will provide a comprehensive explanation of each aspect of improvement. Fig 2 illustrates FB-YOLOv7 network structure.

#### 2.2.1 Effici-Bi-Level Routing Attention

In the practical implementation of helmet detection, the targets on the monitoring image are typically small and closely packed, making them vulnerable to intricate road conditions and weather variations. This study incorporates a self-attention technique to enhance the feature extraction capability of the model and minimise the rate of missed target detection. The self-attention mechanism enhances the network’s performance and efficiency by capturing the correlation between distinct points in the sequence. This allows the network to prioritise the most significant or relevant elements of the image. Nevertheless, the current Swin Transformer [21], ViT [22], CvT [23], and other models suffer from issues such as extensive computational requirements and high memory usage. Therefore, this study introduces an enhanced sparse self-attention mechanism module known as E-BRA (Effici-Bi-Level Routing Attention), based on the BRA (Bi-Level Routing Attention) [24].

**Fig 3:**
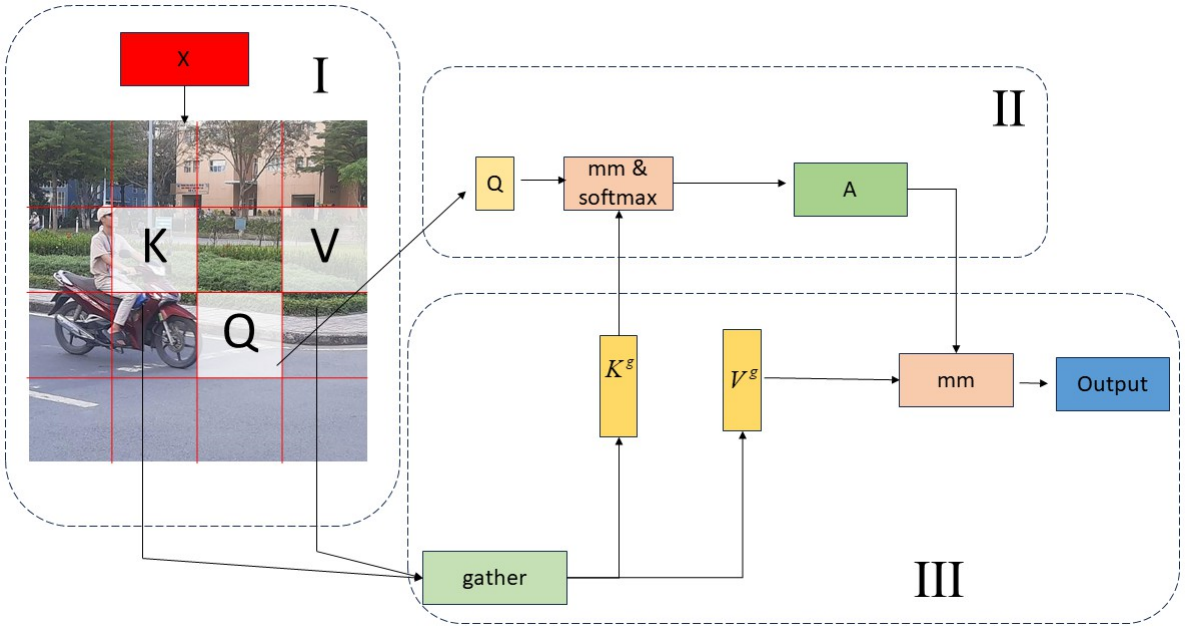
E-BRA structure.

The E-BRA primarily consists of three distinct sections, as illustrated in Fig 3. The initial component involves the partition and input projection within the specified region. The implementation involves dividing the input feature map *X* ∈ *R*^*H*×*W*×*C*^ into *S* × *S* non-overlapping sections and obtaining $Q,K,V\in R^{S^{2}\times \frac{HW}{S^{2}}\times C}$ by linear mapping.

$$Q=X^{T}W^{q}$$ (1)

$$K=X^{T}W^{k}$$ (2)

$$V=X^{T}W^{v}$$ (3)

Where *W*^*q*^, *W*^*k*^ and *W*^*v*^ are the projection weights belonging to query, key, and value, respectively.

The second component involves the region-to-region routing of the graph. The correlation between the two regions can be determined by multiplying the values of *Q* and *K*. Similarly, one can construct the correlation matrix, denoted as *A*^*r*^ that shows the relationship between different regions.


$$A^{r}=Q^{r}{\left(K^{r}\right)}^{T}$$ (4)

In this context, *Q*^*r*^ and *K*^*r*^ represent the matrices that include the average values of the query and key in each region, respectively.

The correlation matrix *A*^*r*^ depicts the interrelationship between regions in the feature map. To obtain the routing index matrix *I*^*r*^, it is necessary to eliminate the portion with low correlation. *I*^*r*^ reflects the most concentrated area following the screening process.

$$k=\left|\left\{(i,j)|A^{r}(i,j)>\lambda_{r}\right\}\right|$$ (5)

$$I^{r}=\text{topkIndex}(A^{r})$$ (6)

where k is the number of regions in *I*^*r*^ and *λ*_*r*_ is the correlation threshold.

The third part is Token-to-Token attention.

$$K^{g}=gather(K,I^{r})$$ (7)

$$V^{g}=gather(V,I^{r})$$ (8)

$$BRA=Attention(Q,K^{g},V^{g})+LE(V)$$ (9)

where gather() refers to pooling and concatenating all the corresponding tensors in the routing index matrix, and LE() is the local enhancement of V by the deep convolutional network.

**Fig 4:**
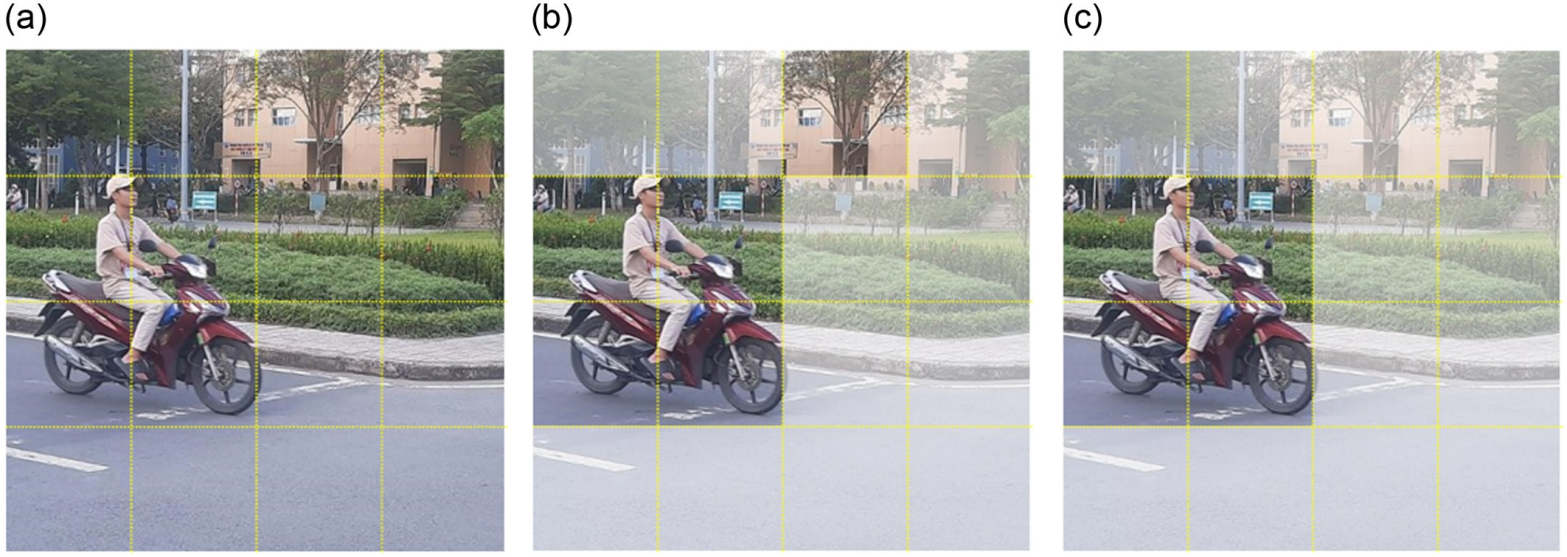
Transformer, BRA, E-BRA feature extraction area.

As depicted in Fig 4, the Transformer uses the complete feature map as its input, leading to the utilisation of a significant amount of computational resources. BRA selectively removes regions that are unrelated to target detection, hence enhancing the efficiency of feature extraction. However, there is a potential risk of eliminating desirable feature regions or preserving low-value feature regions. E-BRA differs from BRA in that it replaces the fixed value of *I*^*r*^ capacity with a dynamic variable that varies with *λ*_*r*_. This allows for *λ*_*r*_ adjustment to maintain a balance between computational resources and feature extraction regions.

To assess the efficacy of the E-BRA module suggested in this paper, the YOLOv7-tiny model is employed to evaluate its merits and drawbacks. Table 1 displays the outcomes of the experiment.

**Table 1:** The parameters of the improved model are compared in task performance.

Model	BRA	E-BRA
mAP_@.5_	85.2	86.6
F1	82.5	84.5
FPS	103	124

According to the data presented in Table 1, the enhanced E-BRA outperforms BRA in terms of both accuracy and F1 score. Furthermore, there has been a substantial enhancement in detection efficiency. The results provide clear evidence of the upgraded E-BRA’s effectiveness in fulfilling the reduced computational resource needs of edge terminal devices.

#### 2.2.2 Asymptotic feature pyramid network

The tiny size of helmets in most photos makes them easy to overlook when using high-level features in helmet recognition applications. While the YOLOv7-tiny model can utilize all feature layers in a fixed manner through PAnet, this approach has drawbacks. It consumes significant computational resources and can lead to suboptimal results due to information loss during transmission. In order to solve this problem, this paper introduces AFPN [25] to realize the fusion of different levels of features.

**Fig 5:**
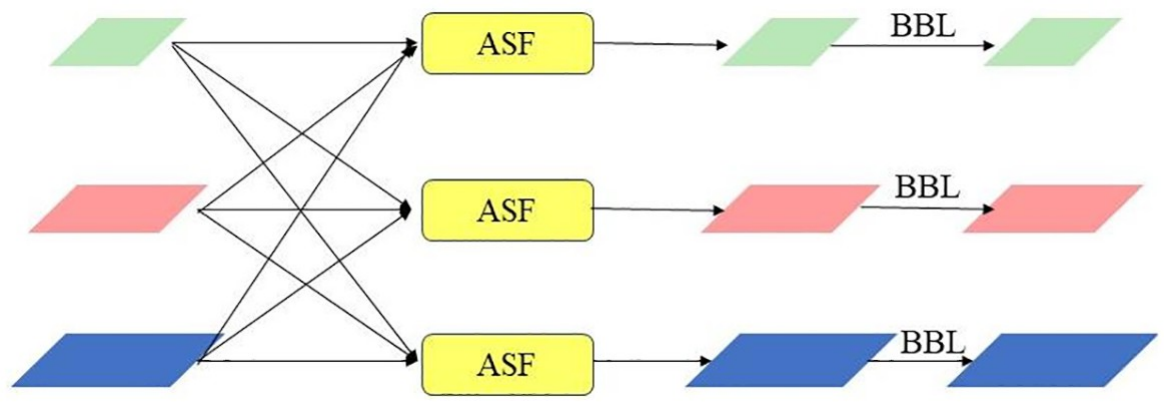
AFPN structure.

Fig 5 illustrates the main implementation of AFPN through the introduction of an ASF module, which adaptively fuses different levels of features through weighted average and alignment. The fusion formula is as follows:

$$y_{ij}^{l}=\alpha_{ij}^{l}\cdot x_{ij}^{1\to l}+\beta_{ij}^{l}\cdot x_{ij}^{2\to l}+\gamma_{ij}^{l}\cdot x_{ij}^{3\to l}$$ (10)

where $\alpha_{ij}^{l}$, $\beta_{ij}^{l}$ and $\gamma_{ij}^{l}$ denote the spatial weights of the features in layer l and the constraints are $\alpha_{ij}^{l}+\beta_{ij}^{l}+\gamma_{ij}^{l}=1$.

The process in AFPN involves the fusion of adjacent features at a low level, followed by the fusion of the resulting features with higher-level features. This fusion method does not directly combine features with a significant disparity in size, hence addressing the semantic gap that exists between non-adjacent layers. This novel progressive fusion technique circumvents the notable disparities in feature fusion across various sizes, efficiently harnesses multi-scale feature data, and preserves the characteristics of each level.

#### 2.2.3 Loss function improvement

At the moment, YOLOv7-tiny typically makes use of the CIoU [26] loss function. Because CIoU considers the bounding box regression’s overlap area, centre point distance, and aspect ratio, its boundary regression loss computation is more precise. The CIoU loss function has the following expression:

$$L_{CI\text{o}U}=1-I\text{o}U+\frac{\rho^{2}(\text{b,}\;\;{\text{b}}^{\text{gt}})}{{\text{c}}^{2}}+\alpha v$$ (11)

$$\alpha =\frac{v}{1-IoU+v}$$ (12)

$$v=\frac{4}{\pi^{2}}{\left(\text{arctan}\frac{\omega^{gt}}{h^{gt}}-\text{arctan}\frac{\omega }{h}\right)}^{2}$$ (13)

where *ρ*^2^(b, b^gt^) denotes the Euclidean distance between the predicted frame and the center point of the real frame, *c* denotes the diagonal distance at the smallest closed region that can contain both the predicted and real frames, *α* enotes the weight coefficient, and *v* measures the consistency of the aspect ratio between the predicted frame and the real frame, *ω*^*gt*^ is the width of the true frame, *h*^*gt*^ is the height of the true frame, *ω* is the width of the predicted frame, and *h* is the height of the predicted frame.

Nevertheless, the CIoU loss function possesses two inherent defects that must not be overlooked. The value domain of the inverse tangent function in the formula for calculating the penalty term in the CIoU loss function is limited to the range of (0, π/2). However, this conflicts with the requirement for numerical normalization. To address this issue, it becomes necessary to introduce new coefficients to achieve normalization, which in turn increases the computational complexity. Furthermore, the penalty term exhibits excessive sensitivity to abnormal situations, leading to a diminished robustness of the penalty term and more pronounced oscillations in the loss value. These two characteristics are particularly noticeable in edge terminal devices that lack significant computing power resources.

This research proposes the adoption of the ECIoU [27] loss function as a more efficient and direct alternative to address the aforementioned issues and compensate for the shortcomings of the CIoU loss function. The ECIoU loss function can be expressed as:

$$L_{ECI\text{o}U}=1-I\text{o}U+\frac{\rho^{2}({\text{b},\;\text{b}}^{\text{gt}})}{{\text{c}}^{2}}+\beta \theta$$ (14)

$$\beta =\frac{\theta }{1-IoU+\theta }$$ (15)

$$\theta ={\left(\frac{1}{1+e^{-\frac{w^{gt}}{h^{gt}}}}-\frac{1}{1+e^{-\frac{w}{h}}}\right)}^{2}$$ (16)

The primary enhancement concept of the ECIoU loss function is to represent the aspect ratio of the actual frame as the domain of the sigmoid function and optimize the penalty term of the loss function using function-based thinking. The penalty term produced in this manner has a value domain of (0, 0.25), which aligns more closely with the requirements of numerical normalization compared to the penalty term that does not account for any loss of the original information. Furthermore, as a result of the characteristics of the sigmoid function, the penalty term θ is more resilient and exhibits a more gradual change compared to the penalized term. Hence, the utilization of the ECIoU regression loss function leads to accelerated convergence, improved localization outcomes, enhanced model performance, and heightened sensitivity towards smaller targets.

**Fig 6:**
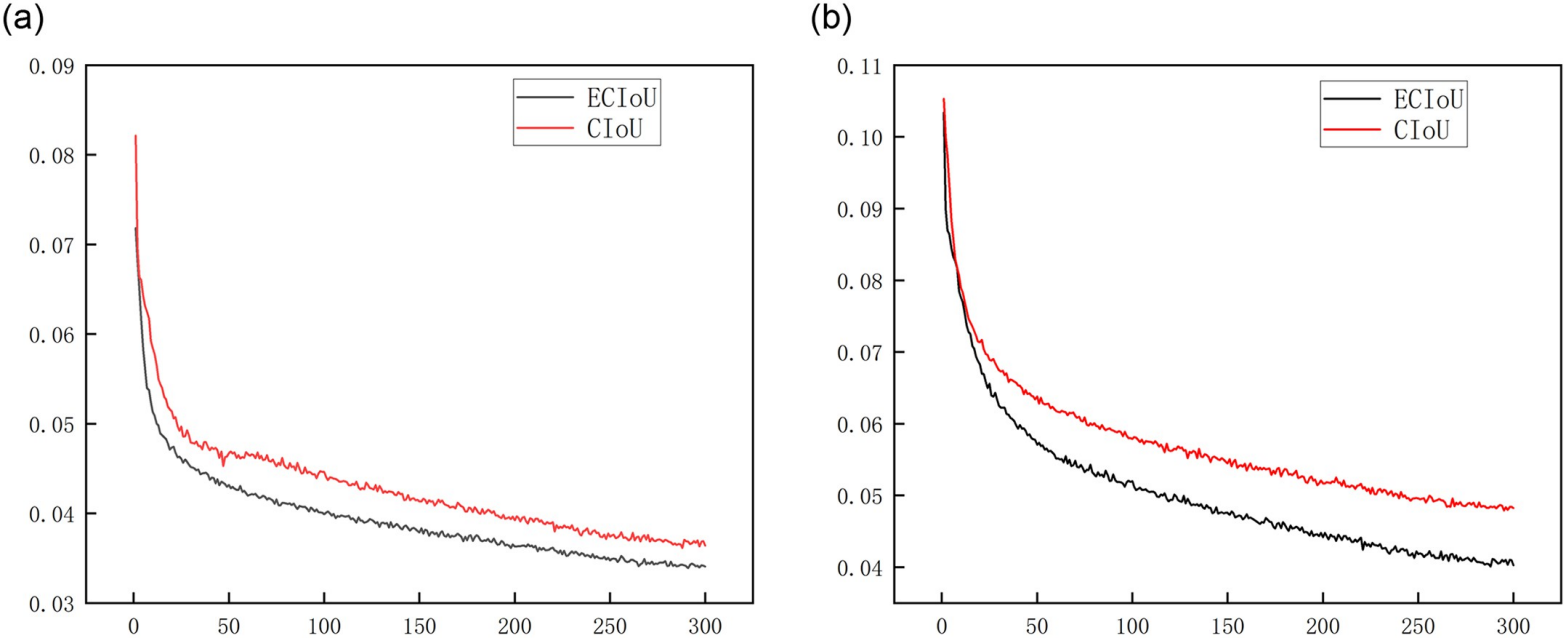
(a) Box loss comparison of loss function. (b) Cls loss comparison of loss function.

The comparison graph of ECIoU and CIoU, shown in Fig 6, clearly demonstrates that the ECIoU curve has a smoother and more consistent trajectory. Furthermore, it reliably generates outputs with lower losses throughout numerous iterations. This highlights the effectiveness of the penalty term in the optimization function of ECIoU.

## 3 Experiment results

### 3.1 Experimental environment

To assure the accuracy of the model, all tests in this study are conducted using identical hardware and software configurations, ensuring that the results are solely attributable to the model itself. The studies were performed on a Windows 11 system environment using an NVIDIA GeForce RTX 3080 Laptop GPU with 16GB of video RAM. The Pycharm software used in this experiment was configured with the following environments: pytorch 2.0.1, python 3.11, and CUDA 11.7.

The experimental training parameters are set as follows: the initial learning rate is 0.0001, the Batch _ size is set to 4, the Image size is 640, the Adam optimizer is selected for optimization, the weight attenuation coefficient is 0.0005, the epoch is 300, and the learning rate momentum parameter is 0.94. The Warmup method is trained, and one-dimensional linear interpolation is used to update the learning rate. After Warmup, the cosine annealing algorithm is used to update the learning rate.

#### 3.1.1 Datasets

This paper focuses on two datasets that involve individuals wearing helmets. One of the datasets is the Helmet detection dataset from Roboflow Universe. It primarily consists of images of bicycles and other two-wheeled vehicles. The dataset contains a total of 4,311 images, which have been re-labelled into five categories: With Helmet, Without Helmet, Motorcycle, Bicycle, and Electric ikes. We have obtained the Daylight-v1 dataset from Roboflow Universe as an additional dataset. The dataset primarily consists of 4,374 images related to driving electric vehicles. These images are categorised into three tags: With Helmet, Without Helmet, and Vehicles. The two data sets are partitioned into a training set, a validation set, and a test set in an 8:1:1 ratio.

#### 3.1.2 Model evaluation

In this paper, common metrics like accuracy rate (precision), recall rate (recall), F1 score, and average accuracy mean value (mAP) are used to objectively compare the model’s pros and cons in detecting the effect of helmet wearing.

$$\text{Precision}=\frac{TP}{TP+FP}$$ (17)

$$\text{Re}call=\frac{TP}{TP+FN}$$ (18)

$$F1=2\cdot \frac{\text{Pre}ci\text{si}on\cdot \text{Recall}}{\text{Pre}ci\text{si}on+\text{Recall}}$$ (19)

Where TP represents the number of correctly predicted positive samples, FP represents the number of incorrectly predicted negative samples, and FN represents the number of incorrectly predicted positive samples.

In this paper, mAP_@.5_ and mAP_@.5:95_ are selected as the evaluation metrics. mAP_@.5_ is the value when the threshold is taken as 0.5, and mAP_@.5:95_ is the average of all the values obtained when the threshold is taken as 0.05 steps from 0.5 to 0.95.

The assessment measures chosen for this paper are mAP_@.5_ and mAP_@.5:95_. The mAP_@.5_ refers to the metric value obtained when the threshold is set at 0.5. On the other hand, mAP_@.5:95_ represents the average of all the metric values obtained when the threshold is incremented by 0.05 steps from 0.5 to 0.95.


$$\text{m}AP=\frac{\sum_{\text{i}=1}^{\text{k}}AP_{\text{i}}}{\text{k}}$$ (20)

Furthermore, to thoroughly assess the performance of the comprehensive model, the quantity of model parameters and the transmission frame rate per second (FPS) are employed as evaluation metrics to gauge the intricacy of the model and the speed of detection, respectively.

### 3.2 Experimental results and analysis

#### 3.2.1 Comparison of Baseline networks

To assess the efficacy of FB-YOLOv7, this study initially evaluates FB-YOLOv7 in comparison with YOLOv7-tiny using the primary evaluation criteria. The outcomes are presented in Table 2, using a classification accuracy criterion of 0.5.

**Table 2:** The overall and classification accuracy of YOLOv7-tiny and FB-YOLOv7 on two datasets.

Dataset	Helmet detection				Daylight-v1			
Model	mAP_@.5_	With Helmet	Without Helmet	Vehicles	mAP_@.5_	With Helmet	Without Helmet	Vehicles
YOLOv7-tiny	84.4	81.8	80.1	86.7	91.2	86.1	85.3	96.3
FB-YOLOv7	87.2	85.3	84.4	88.8	94.6	92	89.8	97.2

Table 2 demonstrate that FB-YOLOv7 surpasses YOLOv7-tiny in terms of accuracy metrics on both datasets. FB-YOLOv7 demonstrates superior performance over YOLOv7-tiny in the mAP_@.5_ metrics on the Daylight-v1 dataset, with a margin of 3.4%. In terms of individual classifications, the improvement in the motorbike classification is not substantial—only 0.9%. However, the helmet classification exhibits a significant improvement of 5.9%. In the Helmet detection dataset, FB-YOLOv7 shows a significant improvement in the more complex multi-class target scenario, with a substantial increase of 2.8% in mAP_@.5_. In the single classification task, there is a noticeable improvement of 2% for motorcycles, 2.3% for bicycles, and 1.9% for electric bikes. Additionally, there are significant boosts of 3.5% and 4.3% for the accuracy of detecting helmets and without helmets, respectively, which were initially less accurate. To sum up, FB-YOLOv7 exhibits superior detection accuracy and excels at accurately identifying small targets.

#### 3.2.2 Ablation experiment

In this study, we conduct experiments using different modules and their combinations on both datasets to evaluate the effectiveness of the proposed enhancements. This allows for a comparison analysis. We employ the same settings during the training phase of all trials to guarantee the precision of the experiments. The outcomes are displayed in Table 3, where A represents E-BRA, B represents AFPN, and C represents ECIoU.

**Table 3:** Ablation experimental data.

Dataset	Helmet detection				Daylight-v1			
Model	Param(M)	mAP_@.5_	F1	FPS	Param(M)	mAP_@.5_	F1	FPS
YOLOv7-tiny	6.01	91.2	87.3	164	6.01	84.4	83.6	161
A	6.16	94.1	91.3	127	6.16	86.6	84.5	124
B	6.13	91.6	90.6	167	6.53	84.9	84.0	165
C	6.01	91.5	89.6	166	6.01	84.6	83.8	161
A+B	6.68	94.3	91.8	129	6.67	86.9	84.7	125
A+C	6.16	94.4	91.3	128	6.16	86.8	84.6	124
B+C	6.13	92.0	91.1	168	6.01	85.1	85.1	167
A+B+C	6.68	94.6	92.1	129	6.67	87.2	85.4	126

FB-YOLOv7 demonstrates a noteworthy enhancement in accuracy by implementing three new modifications to the Daylight-v1dataset, as indicated by its results. Out of all the modules, the E-BRA module has the most significant impact. It enhances the mAP_@.5_ and F1 values by 2.9% and 4%, respectively; however, it does decrease the detection speed. This outcome demonstrates that the E-BRA module is capable of extracting picture characteristics with more efficiency, hence enhancing the network’s expressive capacity. Simultaneously, the AFPN module provides improvements of 0.4% and 3.3% and enhances the detection efficiency. This demonstrates the benefits of the asymptotic feature pyramid in preserving various layers of features. The enhancement of ECIoU-Loss leads to improved performance, resulting in a 0.3% increase in mAP_@.5_ and a 2.3% increase in F1 values without adding further parameters, confirming its usefulness. The empirical findings from the combined implementation of these modules outperform those of a single module, indicating that these modules can operate harmoniously without any contradictions.

On the Helmet detection dataset, the accuracy and F1 value exhibit a modest reduction as the number of labels increases. However, Table 3’s information suggests that all three development suggestions still apply to this specific data set. The utilisation of the E-BRA module resulted in a 2.2% rise in mAP_@.5_ and a 1.2% increase in F1 values. Similarly, the AFPN module led to a 0.5% increase in mAP_@.5_ and a 0.4% increase in F1 values. The improvement in ECIoU-Loss also played a role in enhancing the results. The performance of a multi-module combination surpasses that of a single module, providing further evidence of the resilience of FB-YOLOv7, as observed in the helmet detection dataset. FB-YOLOv7 has demonstrated its capacity to adapt to intricate and variable real-world situations, regardless of whether they involve binary classification or multi-class classification.

To summarise, FB-YOLOv7 has three improvement points that effectively enhance accuracy and F1 value without any conflicts. This optimisation leads to improved overall performance of the model. Additionally, FB-YOLOv7 is highly adaptable and resilient, making it suitable for both two-class and multi-class classification tasks.

#### 3.2.3 Mainstream model performance comparison

**Fig 7:**
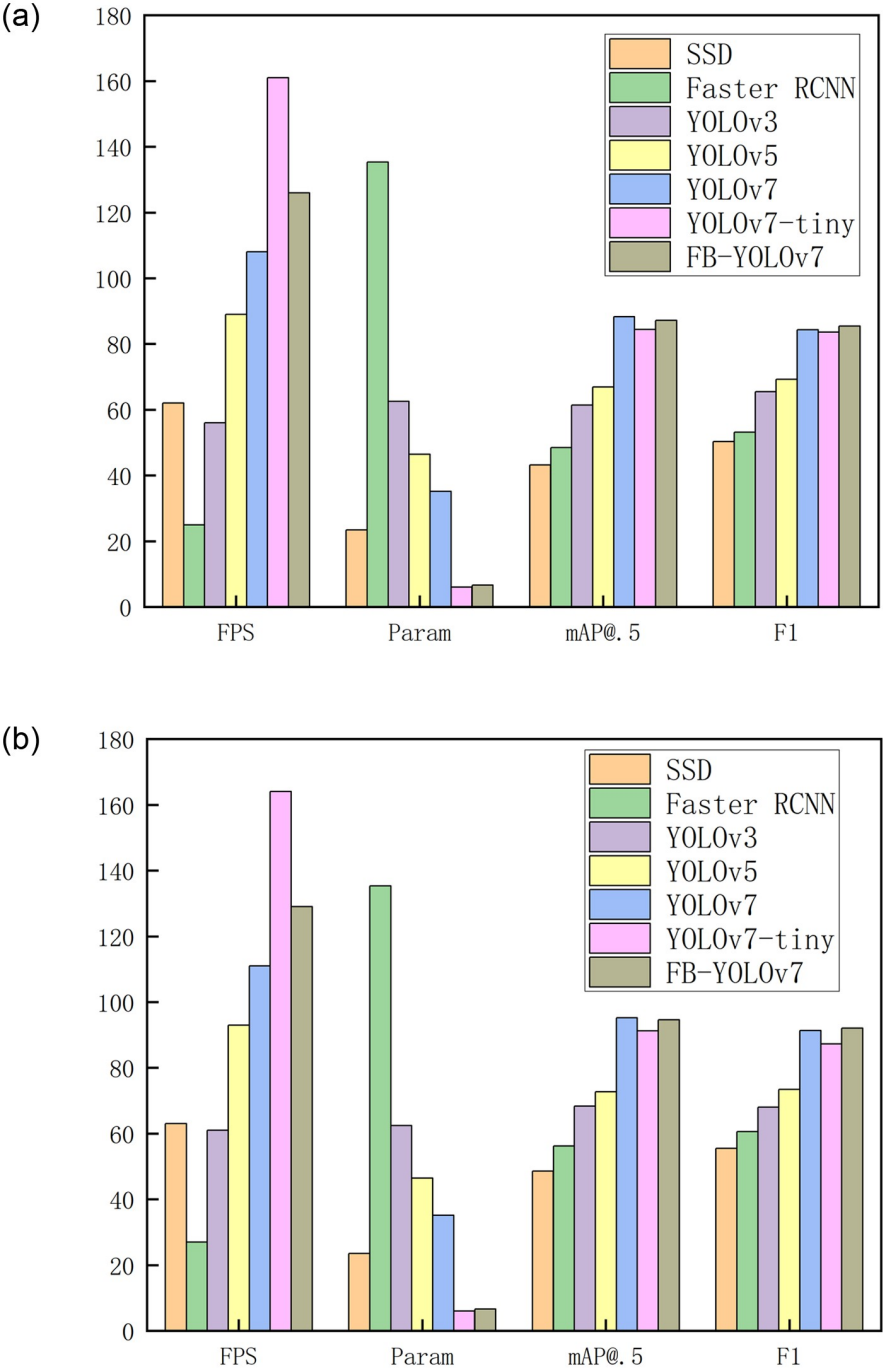
(a) Comparison of mainstream models in Helmet detection (b) Comparison of mainstream models in Daylight-v1.

To ascertain the superiority of FB-YOLOv7 over the existing mainstream detection models, namely Faster RCNN, YOLOv3, YOLOv5, YOLOv7, and YOLOv7-tiny, we have chosen these classical methods to perform tests on datasets for Helmet detection and Daylight-v1. The findings are displayed in Fig 7 and Table 4.

**Table 4:** Experimental data from the mainstream model on each of the two datasets.

Dataset	Helmet detection				Daylight-v1			
Model	mAP_@.5_	F1	FPS	Param(M)	mAP_@.5_	F1	FPS	Param(M)
SSD	43.2	50.3	62	23.4	48.6	55.5	63	23.5
Faster RCNN	48.5	53.2	25	135.3	56.2	60.6	27	135.3
YOLOv3	61.4	65.5	56	62.5	68.3	68.0	61	62.4
YOLOv5	66.9	69.3	89	46.5	72.7	73.4	93	46.5
YOLOv7	88.3	84.3	108	35.2	95.2	91.3	111	35.2
YOLOv7-tiny	84.4	83.6	161	6.01	91.2	87.3	164	6.01
FB-YOLOv7	87.2	85.4	126	6.68	94.6	92.1	129	6.67

Comparing with YOLOv7-tiny, FB-YOLOv7 reduces the FPS by 35 but improves the AP_@.5_ and F1 by 2.8% and 1.8%, respectively. When compared with YOLOv7, FB-YOLOv7 reduces the AP_@.5_ by 1.1% but shows an improvement in the F1 and FPS by 1.1% and 18.1%, respectively. These results indicate that FB-YOLOv7 prioritises the balance between detection speed and accuracy, making it suitable for practical applications with significant practical significance. Furthermore, FB-YOLOv7 possesses comprehensive advantages when compared to other prevalent detection methods. FB-YOLOv7 shows significant improvements in AP_@.5_, F1, and FPS compared to Faster RCNN, YOLOv3, and YOLOv5. Specifically, FB-YOLOv7’s AP_@.5_ is improved by 38.7%, 25.8%, and 20.3%, respectively, while F1 is improved by 32.2%, 19.9%, and 16.1%, respectively. Additionally, FB-YOLOv7 achieves a substantial FPS improvement of 101, 70, and 37 compared to the aforementioned models. The aforementioned conclusions are equally applicable to the dataset used for Daylight-v1. It further demonstrates the superiority of FB-YOLOv7.

By conducting extensive algorithm comparison studies, we can deduce that FB-YOLOv7 demonstrates a substantial enhancement in detection speed while upholding a high level of accuracy. The balance of FB-YOLOv7 enables its effective deployment on edge devices with limited resources. Furthermore, FB-YOLOv7 exhibits significant benefits across several popular detection networks, excelling in both accuracy and speed. This demonstrates its robust potential and broad suitability in real-world application settings. The features of FB-YOLOv7 make it a dependable option for maintaining exceptional performance in many conditions.

#### 3.2.4 Visual comparison

**Fig 8:**
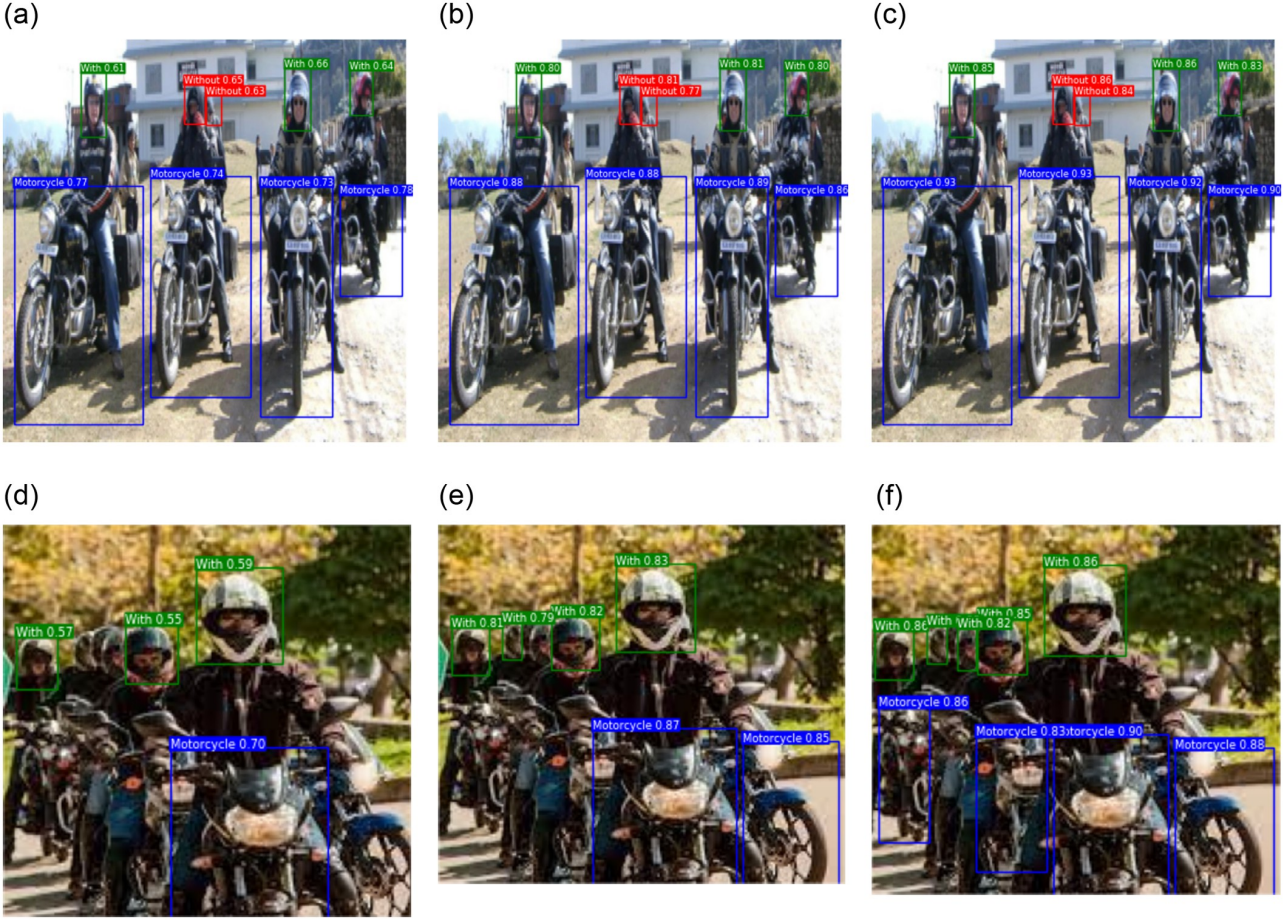
Visualization of detection results for different models.

Fig 8 shows the detection results of YOLOv3, YOLOv5, YOLOv7-tiny, and FB-YOLOv7 in two different situations to show how the improved algorithm works. Upon comparing (a), (b), and (c), it becomes evident that while the benefits of FB-YOLOv7 may not be apparent when detecting large targets that are easy to recognise, the enhanced loss function of FB-YOLOv7 demonstrates exceptional accuracy in recognising helmets that are difficult to identify. When comparing the original image with images (d), (e), and (f), it is evident that the original image contains a high density of small targets, which increases the likelihood of missed detection incidents. While YOLOv3 and YOLOv7-tiny also exhibit some degree of missed detection, FB-YOLOv7 did not miss any detections. FB-YOLOv7 greatly minimises the occurrence of missed detection incidents by utilising the screening and incorporation of global information by E-BRA and the preservation of low-level features by AFPN. To summarise, the enhanced algorithm greatly enhances the capacity to recognise small and obstructed targets while successfully reducing instances of missed detection and incorrect detection. This advancement offers enhanced and dependable technological assistance for a real-time monitoring system.

## 4 Conclusion

This paper introduces a novel network called FB-YOLOv7, designed to accurately recognise the driver’s helmet wearing status and vehicle type. It also addresses the challenge of detecting small targets in helmet detection for two-wheeled vehicles. The algorithm utilises the YOLOv7-tiny framework as its foundation. The original approach incorporates the E-BRA module, AFPN structure, and ECIoU loss function, resulting in a substantial enhancement in the capacity to capture global information and the sensitivity to detect small targets. This research rigorously evaluates the efficacy of FB-YOLOv7 by conducting tests on two datasets: helmet detection and bike helmet detection. Experimental results demonstrate that FB-YOLOv7 minimises the deployment prerequisites and fulfils the criteria for operating on edge terminal devices. Additionally, it exhibits a significant enhancement in both critical accuracy and F1 value, hence affirming its potential for practical applications. To summarise, FB-YOLOv7 has been demonstrated to be effective and can have a significant impact in the domain of helmet detection. However, the network still has ample opportunity for enhancement. In future endeavours, our primary objective should be to gather more extensive data in intricate settings, with a particular emphasis on expediting its detection rate and application in challenging and hostile environments. Additionally, we should strive to enhance network architecture and establish detection schemes with superior functionality and performance.
